# Controlling Enzymatic Activity by Modulating the Oligomerization State via Chemical Rescue and Optical Control

**DOI:** 10.1002/cbic.202100490

**Published:** 2021-10-22

**Authors:** Cosimo Kropp, Astrid Bruckmann, Patrick Babinger

**Affiliations:** ^1^ Institute of Biophysics and Physical Biochemistry Regensburg Center for Biochemistry University of Regensburg 93040 Regensburg Germany; ^2^ Institute of Biophysics and Physical Biochemistry Regensburg Center for Biochemistry University of Regensburg 93040 Regensburg Germany; ^3^ Institute of Biochemistry Genetics and Microbiology Regensburg Center for Biochemistry University of Regensburg 93040 Regensburg Germany

**Keywords:** biocatalysis, chemical rescue, oligomerization, optochemical tools, protein-protein interactions

## Abstract

Selective switching of enzymatic activity has been a longstanding goal in synthetic biology. Drastic changes in activity upon mutational manipulation of the oligomerization state of enzymes have frequently been reported in the literature, but scarcely exploited for switching. Using geranylgeranylglyceryl phosphate synthase as a model, we demonstrate that catalytic activity can be efficiently controlled by exogenous modulation of the association state. We introduced a lysine‐to‐cysteine mutation, leading to the breakdown of the active hexamer into dimers with impaired catalytic efficiency. Addition of bromoethylamine chemically rescued the enzyme by restoring hexamerization and activity. As an alternative method, we incorporated the photosensitive unnatural amino acid *o*‐nitrobenzyl‐*O*‐tyrosine (ONBY) into the hexamerization interface. This again led to inactive dimers, but the hexameric state and activity could be recovered by UV‐light induced cleavage of ONBY. For both approaches, we obtained switching factors greater than 350‐fold, which compares favorably with previously reported activity changes that were caused by site‐directed mutagenesis.

## Graphical abstract

**Exogenous control** of the association state of enzyme complexes can be used to switch activity. A cation‐π bond in the oligomerization interface was disrupted either by a Lys‐to‐Cys mutation or by incorporation of an unnatural amino acid (UAA), resulting in complex disassembly and loss of activity. For switching activity on again, Lys was chemically rescued or the UAA was decaged by light. The complex was reformed and the activity was fully restored.

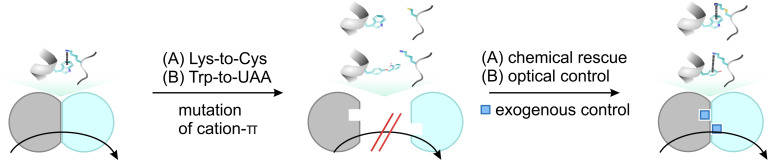

## Introduction

Selective control over enzymatic activity is a longstanding goal in synthetic biology. Especially in the field of biocatalysis, the design of controllable enzymes has been pushed forward in recent years. Engineered enzymatic biocatalysts will replace current production methods of high‐value chemicals for an increased economical and resource efficient production and a decreased environmental impact. Selective control of enzymatic activity for instance helps to organize enzymes in cascades.^[1]^

Control over enzymatic activity can be achieved by a wide variety of techniques such as the implantation of molecular switches. There are various methods to generate such switches, among them chemical rescue and optochemical tools. For so‐called chemical rescue, an essential amino acid residue is mutated to render the enzyme less active or inactive. Upon addition of small exogenous compounds to the purified protein, activity is restored. The compound thereby mimics the mutated residue – in other words, the enzyme becomes dependent on a cofactor. Since its first introduction,^[2]^ several *in vitro* studies using chemical rescue have been performed.^[3]^ Alternative and nowadays more widespread strategies use optochemical tools, which can be subdivided into the overlapping fields of optogenetics^[4]^ and methods that directly control the target protein.^[5]^ For the latter methods, most commonly an unnatural amino acid (UAA) is incorporated into the protein, which is encoded by a reprogrammed stop codon and delivered by a modified tRNA plus aminoacyl‐tRNA synthetase. The sterically challenging properties or other features of the UAA render the enzyme less active. Upon light exposure, the photosensitive UAA is, for example, decaged to the canonical residue it was incorporated for and the protein becomes functional again.

Both for chemical rescue and optical control of enzymes, the most common approaches to switch activity are either based on direct modification of catalytically relevant residues^[6]^ or on indirect blocking of the active site. For example, incorporation of bulky UAAs can impede the access of the substrate to the binding pocket,^[7]^ or introduced and then rescued mutations negatively affect the structural integrity or stability of the protein.^[8]^ Such structure‐based approaches often exploit allosteric effects.^[9]^ Although such approaches seem to be straight forward, it is frequently difficult to achieve activity switching factors >10–100.

Allostery usually includes the interaction of two or more subunits within an enzyme, and many enzymes are at least dimeric proteins. Although the oligomerization state of proteins has already been successfully manipulated by optical control or chemical rescue to control transcription factors^[10]^ or to impede virus‐host interactions,^[11]^ this approach has been scarcely used to directly switch the activity of enzymes. However, it has frequently been reported that the mutational disruption of a functional oligomer is accompanied by a severe decrease of activity or catalytic efficiency, with factors of 10^2^ over 10^5^ to even unlimited, in case the broken complex is completely inactive. Among them are, for example, peroxiredoxins,^[12]^ dihydroorotate dehydrogenase,^[13]^ glutathione transferase,^[14]^ tryptophan synthase^[15]^ or glutamine amidotransferases.^[16]^ Especially high switching factors can be achieved in cases where substrate channeling between the subunits is disrupted, or when two subunits form the active site in concert.^[17]^

**Figure 1:**
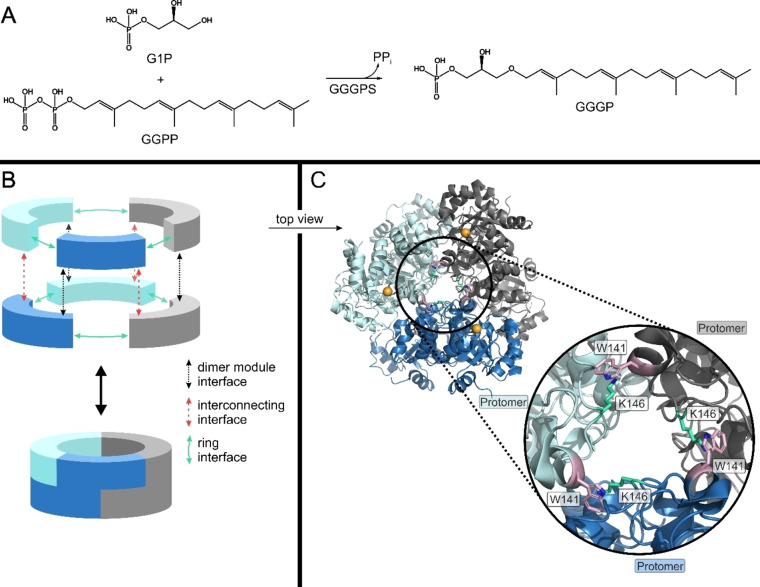
Properties of the GGGPS hexamer. (A) Catalyzed reaction of GGGPS. (B) Schematic visualization of the GGGPS structure in exploded and assembled view. Each building block represents a GGGPS monomer. A dimer module is indicated by matching colors. The figure is adapted from preceding work.^[18]^ (C) Crystal structure of *M. thermautotrophicus* GGGPS (mtGGGPS; PDB ID 4mm1) with co‐crystallized substrate G1P. The ring interface is marked by the black circle. The hot spot residues in the ring interface, W141 (pink) and K146 (green), that form the cation‐π bond between adjacent protomers are shown as sticks. The phosphates of the G1Ps in the upper three protomers are shown as orange spheres.

As a proof‐of‐principle, we set out to switch the activity of geranylgeranylglyceryl phosphate synthase (GGGPS) by modulating its oligomerization state using either chemical rescue or optical control. GGGPS catalyzes the condensation of glycerol 1‐phosphate (G1P) and geranylgeranyl pyrophosphate (GGPP; Figure 1A), which is a key step in the biosynthesis of the typical archaeal membrane ether lipids.^[19]^ Hexameric oligomerization is widely spread among this enzyme family (Figure 1B, C), but can easily be disturbed by introducing mutations at the subunit interfaces. The hexamer then disassembles into dimers,[^19b^, ^20^] which is accompanied by a 380‐fold decrease in catalytic efficiency.^[18]^ We made use of this effect and now disturbed the hexamer either by mutating an essential Lys in the hexamerization interface, which can be chemically rescued in the purified protein, or by inserting the UAA *o*‐nitrobenzyl‐*O*‐tyrosine (ONBY) instead of an essential aromate, which can be optically decaged to a Tyr. With both strategies, we obtained similar switching factors like previously observed in mutational studies with GGGPS, but now by manipulating the purified protein by exogenic means.

## Results and Discussion

We selected the GGGPS from *Methanothermobacter thermautotrophicus* (mtGGGPS) as model enzyme for our proof‐of‐concept study, which allowed us to base on previous results.[^18^, ^19b^, ^20^, ^21^] In the hexameric mtGGGPS, complex formation is mediated by three distinct interfaces: the dimer module interface, the interconnecting interface and the ring interface (Figure 1B). The dimer module interface connects two monomers to a dimer. The other two interfaces assemble the hexamer as a trimer of dimers. A crucial interaction in the ring interface between adjacent dimer modules is a cation‐π bond, which is an interaction of an aromatic residue in the one protomer (Trp in mtGGGPS) and a cationic residue in the other (Lys in mtGGGPS; Figure 1C). Previous studies have confirmed the importance of the cation‐π bond by mutation of either the Trp or the Lys residue. The mutated GGGPS variants disassembled into dimers and showed a massively decreased affinity for the G1P substrate (∼75×) as well as a moderate decrease of the turnover number (∼5×), resulting in a 380× decrease in catalytic efficiency.^[18]^

In a subsequent study, we resurrected evolutionary predecessors of mtGGGPS by ancestral sequence reconstruction.^[20]^ We identified two sequential GGGPS ancestors that were located on the same evolutionary path, called AncGGGPS2_N4 (N4) and AncGGGPS2_N12 (N12). N4 was dimeric and non‐functional, in contrast to hexameric and active N12 (Figures S1–S3, Table S1). We managed to hexamerize N4 by transplanting the ring interface from N12 into N4. The resulting variant AncGGGPS2_N4_IF_n12 (N4_IF_n12), which differs only in five contact interface residues from N4,^[20]^ showed comparable activity as N12 (Figure S7E). Due to the inactivity of N4, N4_IF_n12 promised the possibility to establish an infinite switching factor by modulating its oligomerization state using a single mutation. Hence, in addition to mtGGGPS, we selected N4_IF_n12 as a second study object. For simplicity, we call this variant “RGGGPS” (rescued GGGPS) from now on.

### Cation‐π interaction as molecular switch

Cation‐π interactions are widespread among oligomers. They occur in the subunit contact interfaces from about 50 % of all protein complexes and significantly contribute to their stabilization.^[22]^ Cation‐π interactions are accessible to manipulation by both chemical rescue or optical control. The aromatic residue can be rescued by non‐covalent binding of indole after its mutation to a small residue.^[9a]^ The cationic counterpart, if it is a Lys, it can be rescued by bromoethylamine (BrEtAm) after its mutation to Cys, forming a covalently linked Lys‐mimetic residue.^[6b]^ Alternatively, both interacting residues can be replaced by sterically demanding photocaged UAAs: the aromate by *o*‐nitrobenzyl‐*O*‐tyrosine (ONBY), which can be photo‐decaged to Tyr, the cationic counterpart by photocaged Lys.^[23]^

**Figure 2:**
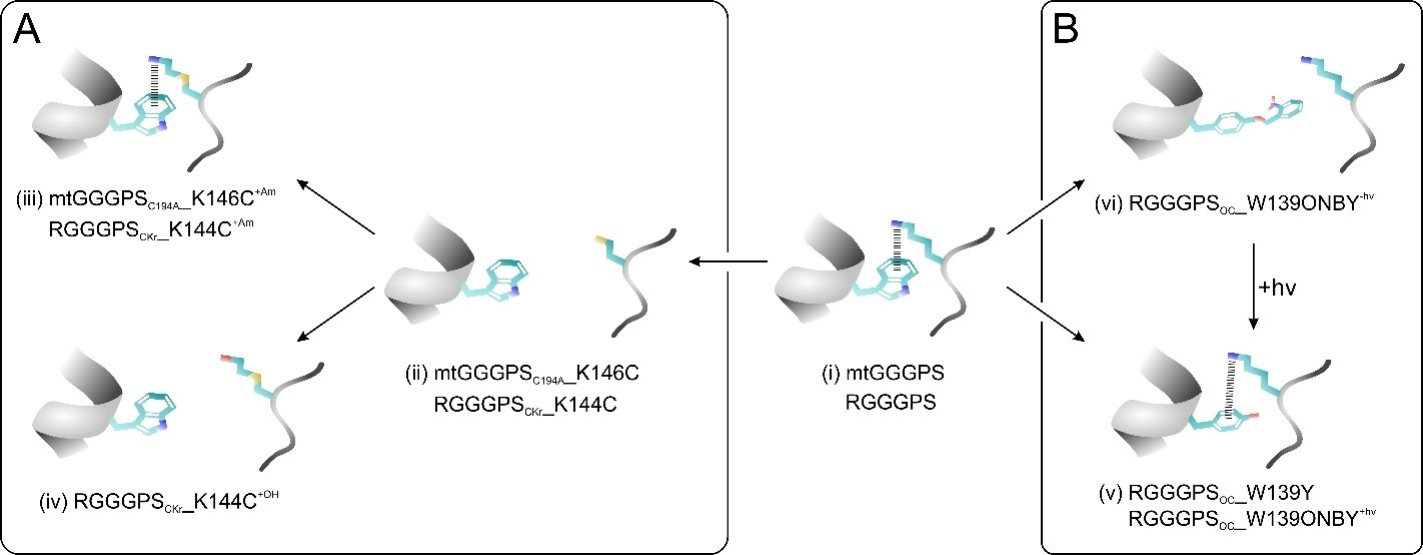
Switch conceptualization. Starting from mtGGGPS or RGGGPS with a Trp‐Lys cation‐π interaction, two strategies were implemented. (A) Chemical rescue; (B) Optical control. For detailed description, see the text. An intact cation‐π interaction is symbolized by black bars between the aromatic and the cationic moiety. The indices mean: Am, bromoethylamine; OH, bromoethanol; CKr, Chemical lysine (K) rescue; OC, optical control; C194A, auxiliary mutation. Detailed chemical structures of the modified amino acids are shown in Figure S4.

We followed both approaches, which are depicted in Figure 2A (chemical rescue) and Figure 2B (photocontrol), respectively. In mtGGGPS, the cation‐π interaction is formed by the residues W141 and K146, in RGGGPS by W139 and K144 (Figure 2(i)). To begin with, we pursued chemical rescue for mtGGGPS, which first required to remove a native Cys to avoid inadvertent effects (introduced mutation C194A). The cation‐π interaction was then disrupted by mutating the Lys to a Cys (K146C). Similarly, we proceeded with RGGGPS and introduced the analogous mutation K144C (Figure 2(ii)). After incubation with BrEtAm (Figure 2(iii)), the cation‐π bond was intended to be re‐established. As control, RGGGPS_CKr__K144C was supplemented with bromoethanol (BrEtOH). BrEtOH generates an isosteric alcohol instead of an amine after Cys modification and should therefore not be able to rescue the cation‐π interaction (Figure 2(iv)). Optical control was only implemented for RGGGPS by ONBY incorporation instead of the aromate (W139ONBY; Figure 2(v)). This inactive variant is intended to be reactivated by light (+hν; Figure 2(vi)), which decages ONBY to Y. As control, the Trp was directly mutated to Tyr (W139Y; Figure 2(vi)).

### Activation of catalytic activity by switching the oligomerization state

**Figure 3:**
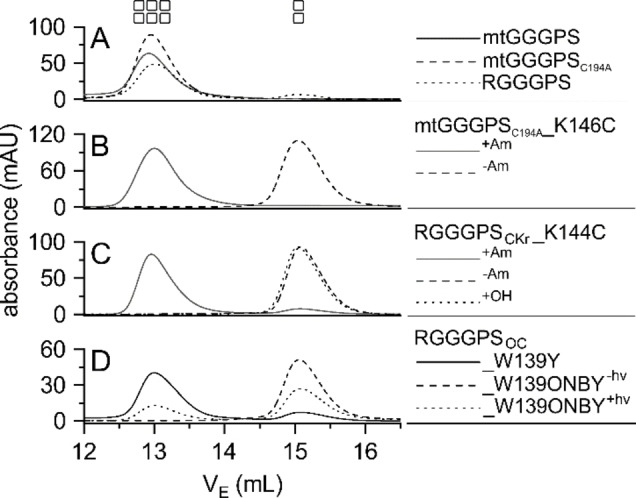
Oligomerization states of GGGPS variants. The denoted proteins (40 μM subunit concentration) were applied to a S200 10/300 GL analytical column, which was equilibrated with 50 mM potassium phosphate, pH 7.5, 300 mM KCl. Elution was performed at a flow rate of 0.4 mL min^−1^, followed by measuring the absorbance at 280 nm, and plotted against the elution volume. The derived oligomerization states are indicated by rectangles (2=dimer, 6=hexamer) and listed in Table S1.

We expressed the variants depicted in Figure 2 in *E. coli* and purified the proteins by immobilized metal affinity chromatography (IMAC). A preparative size exclusion chromatography (SEC) purification step was added to remove residual contaminating proteins of higher and lower molecular weight, which facilitated subsequent analysis of switched oligomerization states. Before and after treating the proteins for chemical rescue or with light, we subjected them to analytical SEC in combination with static light scattering (SLS) to determine the oligomerization states of the complexes and to obtain exact molecular masses. As expected, wild type mtGGGPS and mtGGGPS_C194A_ as well as RGGGPS eluted as hexamers (Figure 3A). For chemical rescue, mtGGGPS_C194A__K146C and RGGGPS_CKr__K144C were incubated over night with 20 mM BrEtAm (^+Am^) or without (^−Am^). The Lys‐to‐Cys mutation led to exclusively dimeric proteins in both mtGGGPS_C194A__K146C^−Am^ and RGGGPS_CKr__K144C^−Am^. For both proteins, BrEtAm rescued the hexameric oligomerization state to almost 100 % (Figure 3B, C). The presence of the Lys‐mimetic residue (Lys‐γ‐S) in RGGGPS_CKr__K144C^+Am^ was verified by tryptic digest coupled with mass spectrometry (Figure S5A). The control RGGGPS_CKr__K144C^+OH^, which was incubated with BrEtOH instead of BrEtAm, remained dimeric (Figure 3C). All oligomerization states and the molecular masses as derived by SLS are summarized in Table S1.

Next, we analyzed the GGGPS variants that were designed for optical control. Because it is a common issue that protein preparations with an integrated UAA contain wild‐type protein contaminations, the preparative SEC purification step can additionally support the homogenous preparation of correctly produced protein, as the incorporation of the UAA changes its oligomerization state, according to our conceptual design. But for RGGGPS_OC__W139ONBY^−hν^, incorporation worked very well, an elution peak corresponding to an hexameric fraction was not detectable in preparative SEC (data not shown). Consequently, the protein eluted as a pure dimer in subsequent analytical SEC, indicating a complete disruption of the hexamer due to the incorporated ONBY (Figure 3D). Incorporation of ONBY instead of W139 was verified by tryptic digest coupled with mass spectrometry (Figure S5B). After the protein was decaged by irradiation at 365 nm for 3 min (RGGGPS_OC__W139ONBY^+hν^), approximately 30 % of it (as estimated from the peak integrals) eluted as a hexamer in SEC, which indicated that the hexamer could be reconstituted as projected. Because ONBY is decaged by light to Tyr, we created the variant RGGGPS_OC__W139Y as control, which eluted almost completely as hexamer under the given experimental conditions (Figure 3D).

To test whether the switch in oligomerization state affected enzymatic activity, we tested the variants with a photometric assay that detects the pyrophosphate which is liberated in the GGGPS reaction (Figure 1A). As expected from *a priori* knowledge from N4 (cf. first paragraph of results; Figure S3), RGGGPS_CKr__K144C^−Am^ showed no activity at all when we incubated the enzyme with saturating concentrations of G1P for 24 h (Figure S6, white triangles). In contrast, after addition of BrEtAm, RGGGPS_CKr__K144C^+Am^ showed rising activity with time. Full restoration of activity was reached after ∼30 min and stayed unchanged even after 24 h of incubation with BrEtAm (Figure S6, black circles).

To determine activity switching factors of the enzymes upon chemical rescue and optical control, we determined the steady‐state kinetic parameters of the variants (Figure S7, Table 1). The variant mtGGGPS_C194A_, which was created as “wild‐type reference” to avoid inadvertent effects in the chemical rescue experiment due to the native Cys, showed similar catalytic parameters like mtGGGPS. In comparison, the variant mtGGGPS_C194A__K146C^−Am^ with disrupted cation‐π interaction and dimeric oligomerization state showed an 8x decrease in k_cat_, a 19x increase in K_M_ and consequently a 160x decrease in k_cat_/K_M._ Within the range of experimental fluctuation, this is consistent with the previously published kinetic data for hexameric mtGGGPS and the dimeric mutant mtGGGPS_W141A (5x decrease in k_cat_, 75x increase in K_M_, 380× decrease in k_cat_/K_M_).^[18]^ After chemical rescue with BrEtAm (mtGGGPS_C194A__K146C^+Am^), activity was fully restored. The rescued variant even showed a slightly elevated activity, corresponding to an activation factor of 350x in terms of k_cat_/K_M_. Because we strived to achieve high switching factors, we analyzed the variant RGGGPS, which is about 25x less active than the wild‐type mtGGGPS, in terms of k_cat_/K_M_. As discussed above, RGGGPS_CKr__K144C^−Am^ showed no detectable activity any more. After chemical rescue with BrEtAm (RGGGPS_CKr__K144C^+Am^), activity was fully restored again, which is tantamount to an infinite switching factor.

**Table 1:** Catalytic parameters of GGGPS variants in inactive and active states.^[a]^

Protein	k_cat_ (s^−1^)	K_M_ (μM)	k_cat_/K_M_ (s^−1^ M^−1^)
mtGGGPS	2.3 (±0.05)×10^−1^	2.0±0.3	11.5 (±1.7)×10^4^
mtGGGPS_C194A_	2.9 (±0.1)×10^−1^	3.3±0.6	8.8 (±1.8)×10^4^
mtGGGPS_C194A__K146C^+Am^	2.3 (±0.04)×10^−1^	1.2±0.1	19.2 (±2.4)×10^4^
mtGGGPS_C194A__K146C^−Am^	3.5 (±0.07)×10^−2^	63.4±5.8	5.5 (±0.6)×10^2^
RGGGPS	4.4 (±0.2)×10^−2^	14.2±2.2	3.6 (±0.7)×10^3^
RGGGPS_CKr__K144C^+Am^	3.7 (±0.05)×10^−2^	6.7±0.5	5.8 (±0.5)×10^3^
RGGGPS_CKr__K144C^−Am^	–	–	–
RGGGPS_OC__W139Y	3.8 (±0.09)×10^−2^	10.2±1.2	3.7 (±0.5)×10^3^
RGGGPS_OC__W139ONBY^+hv^	2.2 (±0.07)×10^−2^	22.5±3.0	0.99 (±0.2)×10^3^
RGGGPS_OC__W139ONBY^−hv^	–	–	–

[a] Kinetic parameters were determined at 40 °C with a photometric assay for phosphate detection in duplicates and fitting the Michaelis‐Menten equation to the data (Figure S7). Standard deviations are given. – no analyzable activity in the presence of 500 nM protein, 250 μM G1P and 11 μM GGPP.

Analogously, we investigated reactivation upon ONBY decaging to Tyr in the photosensitive variant RGGGPS_OC__W139ONBY. Again, RGGGPS_OC__W139ONBY^−hν^ was non‐functional (Figure S3). Upon decaging, 27 % of catalytic efficiency were restored in comparison to RGGGPS. This is congruent with the portion of about 30 % hexameric protein after light treatment (Figure 3D). The reason for this incomplete restoration of the active hexamer most likely is that frequently, a large portion of ONBY is irreversibly reduced in cells and thus becomes uncleavable.^[9b]^ We could detect the presence of the reduced form by tryptic digest coupled with mass spectrometry also in our preparation (Figure S5C). The control RGGGPS_OC__W139Y showed almost identical catalytic parameters as RGGGPS.

### Physico‐biochemical analysis of the inactive and reactivated variants

The structural integrity of all variants used in the study was monitored by circular dichroism (CD) spectroscopy. All spectra indicated a well‐defined secondary structure with no significant differences between active, inactive and reactivated state (Figure S8). This supports that the disruption of the hexamer is not associated with larger secondary structure rearrangements, but mainly results from the destruction of the cation‐π interaction. Nevertheless, we assume that the significant loss of catalytic efficiency upon hexamer disruption is caused by small structural rearrangements that affect substrate binding. The oligomerization interface with the disturbed cation‐π interaction is not immediately neighboring the active site (Figure 1),[^18^, ^19b^] but especially K_M_ is drastically impaired in the dimeric variants (Table 1). To shed light on this, we analyzed binding of the substrate G1P to RGGGPS_CKr__K144C^−Am^ and RGGGPS_CKr__K144C^+Am^ by isothermal titration calorimetry (ITC) (Figure S9). Although a dissociation constant could not be derived from the raw data due to the high protein concentrations that were necessary to obtain good signals, the data clearly indicated a severely impaired binding of G1P to RGGGPS_CKr__K144C^−Am^ which could be rescued by addition of BrEtAm.

We have shown previously that modulation of the oligomerization state of mtGGGPS influences the thermal stability of the protein. While the overall fold of mtGGGPS is extremely thermostable, activity is lost in the dimeric variants at much lower temperature than in the hexamer, and we assume this is due to an extra stabilization of the active site in the hexamer.^[18]^ We tested by nano differential scanning fluorimetry (nanoDSF) whether this is also the case for the inactive and rescued mtGGGPS variants. mtGGGPS_C194A_ shows a very high thermal stability with a melting temperature >95 °C like mtGGGPS (Figure S10A; no visible peak in the first derivative plot of nanoDSF). Like previously reported for dimeric mtGGGPS_W141A,^[18]^ mtGGGPS_C194A__K146C^−Am^ shows a reduced thermal stability with a transition at approx. 62 °C, which can be completely restored to wild‐type level in mtGGGPS_C194A__K146C^+Am^ (Figure S10B). The tested RGGGPS variants all show very similar thermal denaturation in nanoDSF with a transition at 88–92 °C (Figure S10C, D). Due to the strong fluorescence of ONBY, RGGGPC_OC__W139ONBY could not be analyzed by nanoDSF.

In summary, these results support that activity switching of GGGPS by modulating its oligomerization state is accompanied by changes in the physico‐biochemical properties of the enzyme that can be robustly monitored and controlled. As expected, some structural properties like thermal stability are impaired in some inactivated variants, but can be restored to wild‐type level upon exogenous reactivation.

## Conclusion

Our proof‐of‐concept study demonstrates that catalytic activity of our model enzyme, GGGPS, can be readily and efficiently regulated *in vitro* by changing the association state via chemical rescue or photo‐switching. It is obvious that chemical rescue with BrEtAm is not a suitable approach for *in vivo* applications due to its toxicity, and also incorporation of UAAs remains challenging for use in living cell systems. But both methods have been well established over the years and might work well for production of switchable enzymes for the *in vitro* synthesis of chemicals, such as in one‐pot reactions. Our central aim, however, was to demonstrate that the control of the oligomerization state by introducing molecular switches might be a widely applicable method to control enzymatic activity of homo‐ or heteromeric enzyme complexes, since drastic changes of activity upon oligomer disruption are common and frequently reported in the literature. Importantly, switching activity by reversibly dissociating an oligomer using externally added chemical compounds or UAAs has several advantages compared to the direct modification of the active site. For example, it is often straightforward to dissociate oligomers by single mutations, e. g. by incorporation of bulky photocaged amino acids or by disrupting cation‐π interactions. The latter are common in contact interfaces and represent ideal targets to establish a switch, because both interaction partners are accessible to chemical rescue and optical control. Furthermore, the change in oligomerization state is predictable, rather easy to detect and to be analyzed by SEC, and it allows high‐quality purification of either switched state for *in vitro* use of enzymes.

We additionally used the benefit of ancestral sequence reconstruction (ASR) in our study, which usually creates a bundle of homologous proteins with diverse features concerning activity. A main advantage of ASR‐generated protein variants is that they are frequently very stable in structure, not rarely even more stable than the extant representatives.^[24]^ This allowed us either to select highly active variants in the on state (the extant wild‐type variants) with a good activity switching factor (>100×) to the off state, or variants that are completely inactive in the off state albeit with less activity in the on state, just as the application conditions would require.

## Experimental Section

**Cloning and site‐directed mutagenesis**: All mutated variants as described in Table S1 were generated by QuickChange mutagenesis^[25]^ with oligonucleotides listed in Table S2. For ONBY incorporation, a stop codon point mutation (TAG=*amber*) was introduced into RGGGPS (previously called AncGGGPS2_N4_IF_n12^[20]^). To confirm the successful mutation, all genes were sequenced entirely. RGGGPS has been cloned previously,^[20]^ as well as mtGGGPS.^[19b]^ The sequence numbering for mtGGGPS used in this study refers to EMBL ENA entry AAB85058.

**Production and purification of proteins**: Proteins were produced by heterologous gene expression in BL21‐Gold(DE3) *E. coli* cells (Agilent Technologies). Transformed cells were grown at 37 °C in LB medium containing ampicillin (150 μg mL^−1^) until an OD_600_ of 0.6. Expression was induced by adding 1 mM isopropyl‐β‐D‐1‐thiogalactopyranoside (IPTG) and growth was continued overnight at 20 °C. Cells were harvested by centrifugation and suspended in 50 mM Tris, pH 8.0, 300 mM NaCl, 10 mM imidazole. For the chemical rescue variants (RGGGPS_CKr__K144C, mtGGGPS_C194A__K146C), 10 mM 2‐mercaptoethanol was added to the lysis buffer. Cells were disrupted by sonication and the His‐tagged proteins were purified from the clarified cell extract by immobilized metal chelate affinity chromatography (IMAC) using an ÄKTApurifier system with a HisTrap FF crude column (5 mL, Cytiva). Proteins were eluted by a linear gradient of imidazole (10–500 mM) in 50 mM Tris, pH 8.0, 300 mM NaCl. Contaminating proteins of higher and lower molecular weight, imidazole and salt were removed by subsequent preparative size exclusion chromatography (SEC) on a Highload^TM^ 26/600 Superdex^TM^ S200 pg column (Cytiva) at a flow rate of 1.5 mL min^−1^. The column was equilibrated with 50 mM Tris, pH 8.0. 1 mM Tris(2‐carboxyethyl)phosphine (TCEP) was added for the chemical rescue variants.

The photosensitive variant, RGGGPS_OC__W139ONBY, was also produced by heterologous gene expression in BL21‐Gold(DE3) *E. coli* cells (Agilent Technologies). Chemically competent cells were co‐transformed with the expression vector, carrying the desired TAG codon for ONBY incorporation at position W139, and pEVOL_ONBY. Transformed cells were grown at 37 °C in 6 L LB medium containing ampicillin (150 μg mL^−1^) and chloramphenicol (30 μg mL^−1^) until an OD_600_ of 0.6. Cells were harvested by centrifugation at room temperature and suspended in 600 mL terrific broth (TB) medium. Cell growth was continued at 37 °C to an OD_600_ of 10. Protein production and incorporation of ONBY was induced by addition of 1 mM ONBY, 0.02 % L‐arabinose and 0.5 mM IPTG. Protein production was performed overnight at 20 °C. Cells were harvested by centrifugation, suspended in 50 mM Tris, pH 8.0, 300 mM NaCl, 10 mM imidazole and disrupted by sonication. The His‐tagged protein was purified from cell extract by IMAC in 50 mM Tris, pH 8.0, 300 mM NaCl, applying a linear gradient of imidazole to elute the protein, as described above. Higher oligomers, imidazole and salt were removed by preparative SEC. The column was equilibrated with 50 mM Tris, pH 8.0 and 300 mM NaCl and the system was operated at a flow rate of 1.5 mL min^−1^. All purification steps and all systems were operated in the dark.

Protein concentrations were determined by absorbance spectroscopy or with Bradford assay in case of the photosensitive variant. The molar extinction coefficients ϵ_280_ and the molecular weight were calculated from the amino acid sequence using ProtParam.^[26]^ Proteins were dropped into liquid nitrogen and stored at −80 °C. High concentrations were achieved by concentrating the sample in ultrafiltration units (Amicon Ultra‐15, 10 kDa WMCO, Merck KGaA).

**Selective activation of enzymatic activity**: Chemical rescue of RGGGPS_CKr__K144C and mtGGGPS_C194A__K146C was conducted by supplementing 40 μM protein with 20 mM bromoethylamine (BrEtAm) or bromoethanol (BrEtOH) (final concentrations) in 50 mM Tris, pH 8.0. Both chemicals were purchased from Merck KGaA. Samples were incubated, when not stated otherwise, for 24 h at 40 °C and shaking (300 rpm). Prior to ITC and nanoDSF experiments, buffer solution was exchanged to remove excess BrEtAm using ultrafiltration units (Amicon Ultra‐15, 30 kDa WMCO, Merck KGaA).

Decaging of ONBY was achieved by irradiating the protein mixture in a 1.5 mL reaction tube for 3 min at 365 nm using a high‐power LED (LED Engin, Osram; settings: 700 mA and 16 V). This was done directly before subjecting the protein to the analytical methods in the respective buffer solutions and at the protein concentrations needed for analysis.

**Characterization of the oligomerization state of proteins**: Oligomerization states were determined by SEC experiments, using a calibrated Superdex 200 Increase 10/300 GL column (Cytiva), which was operated in 50 mM potassium phosphate, pH 7.5, 300 mM potassium chloride at a flow rate of 0.4 mL min^−1^ at room temperature (approx. 23 °C). 100 μl of protein with a subunit concentration of 40 μM was applied.

**Molecular weight determination by static light scattering (SLS)**: SLS was performed for molecular weight calculation. Protein (40 μM, subunit concentration) was applied in a volume of 50 μL. A Superdex 200 Increase 10/300 GL column (Cytiva) was operated on an ÄKTAmicro system (Cytiva) in combination with a Viscotek TDA 305 triple detector array (Malvern) including right‐angle light scattering (RALS) and refractive index (RI) detectors. The system was operated in degassed buffer (50 mM potassium phosphate, pH 7.5, 300 mM KCl) at a flow rate of 0.3 mL min^−1^ at room temperature (approx. 23 °C). Data was analyzed using the OmniSec software (Viscotek, version 4.7.0; Malvern).

**Steady‐state enzyme kinetics**: The kinetic parameters of the proteins were determined in a G1P dependent continuous enzyme‐coupled assay for phosphate detection.^[27]^ The assay mixture was composed of 50 mM Tris, pH 8.0, 10 mM MgCl_2_, 0.2 % Tween80, 11 μM GGPP, 1.25 mM inosine, 0.027 U mL^−1^ *E. coli* pyrophosphatase (PPase), 0.25 U mL^−1^ bacterial purine nucleoside phosphorylase (PNPase), and 2.5 U mL^−1^ microbial xanthine oxidase (XOD) (all enzymes were obtained from Sigma‐Aldrich). G1P (0.75–1200 μM) was mixed with the assay mixture in a total volume of 200 μL and incubated at 40 °C. The reaction was started by addition of the enzyme (100 nM for mtGGGPS variants and N12, 500 nM for N4 and RGGGPS variants) and followed at 293 nm using a Jasco V650 spectrophotometer using a 1 cm cuvette. The ϵ of uric acid was considered equal to 12.6×10^3^ M^−1^ cm^−1^ at 293 nm. The reaction velocities were calculated from the initial slopes and the protein concentration. Kinetic constants were deduced by fitting the Michaelis‐Menten equation to the data from duplicate measurements using SigmaPlot 13.0. Kinetic and statistic parameters were calculated using the “XY replicate” feature of SigmaPlot.

**Circular dichroism (CD) spectroscopy**: Proteins were diluted to 6 μM in 50 mM potassium phosphate, pH 7.5. CD spectra were recorded from 180–260 nm with a response time of 0.5 sec at a scan rate of 50 nm min^−1^ and 25 °C in a JASCO J‐815 spectrophotometer using a 0.1 cm cuvette. Data was normalized to obtain the mean residue ellipticity, as described in the literature.^[28]^

**Differential scanning fluorimetry (nanoDSF)**: nanoDSF was performed using an excitation power at 280 nm of 20 % for mtGGGPS variants and 60 % for RGGGPS variants. mtGGGPS (20 μM) or RGGGPS (40 μM) protein (subunit concentration) was heated in 50 mM potassium phosphate, pH 7.5 from 20 °C to 95 °C at a ramp rate of 1 K min^−1^ in a Prometheus NT.48 instrument (NanoTemper Technologies GmbH; access provided by 2bind GmbH). Emission was measured at 330 and 350 nm. The change in the ratio of the fluorescence signal at 350 nm to 330 nm with raising temperature was followed. Fluorescence data were fitted by the program supplied by the manufacturer and the apparent midpoint temperature (T_Mapp_) was determined as an operational measure of protein stability. Results are shown as the first derivative of the fluorescence ratio with respect to temperature. Measurements were done in triplicates, which overlapped perfectly.

**Isothermal titration calorimetry (ITC)**: To investigate G1P binding to the chemically rescued RGGGPS_CKr__K144C variant, a MicroCal PEAQ‐ITC microcalorimeter (Malvern Instruments) was used. Degassed protein (150 μM subunit concentration) was added to the analyte cell (280 μL) and a G1P (1 mM) solution was prepared from the identical buffer batch the protein was prepared in (50 mM Tris, pH 8.0, 10 mM MgCl_2_). The G1P solution was titrated to the protein in 2 μL aliquots for a total of 18 injections at 2.5 min intervals at 25 °C during continuous stirring. Titrations of buffer with buffer, protein solution with buffer and buffer with ligand solution were performed as controls. Each titration was baseline corrected. The experimentally observed signals for ligand binding experiment were corrected for the signals of the control experiments. The differential power (DP) between reference and sample cell, necessary to maintain the temperature difference at zero, was plotted against time.

**Tryptic digest and mass spectrometry**: RGGGPS_CKr__K144C^+Am^ and RGGGPS_OC__W139ONBY^−hv^ were run on a 12.5 % SDS‐PAGE gel and were stained with Coomassie G250 (SimplyBlue SafeStain, Lifetech). Protein bands were cut out and subsequently washed with 50 mM NH_4_HCO_3_, a 50 mM NH_4_HCO_3_/acetonitrile mixture (3 : 1), and a 50 mM NH_4_HCO_3_/acetonitrile mixture (1 : 1) and eventually lyophilized. After a reduction/alkylation treatment of cysteines and additional washing steps, proteins were digested in‐gel by trypsin (Trypsin Gold, mass spectrometry grade, Promega) overnight at 37 °C. The resulting peptides were extracted with 50 mM NH_4_HCO_3_ and 50 mM NH_4_HCO_3_ in 50 % acetonitrile. After lyophilization, peptides were reconstituted in 20 mL of 1 % TFA and separated by reversed‐phase chromatography. An UltiMate 3000 RSLCnano System (Thermo Fisher Scientific, Dreieich, Germany) equipped with a C18 Acclaim Pepmap100 preconcentration column [100 μm (inside diameter)×20 mm, Thermo Fisher Scientific] and an Acclaim Pepmap100 C18 nano column [75 μm (inside diameter)×250 mm, Thermo Fisher Scientific] was operated at a flow rate of 300 nL min^−1^ and a 60 min linear gradient of 4 % to 40 % acetonitrile in 0.1 % formic acid. The liquid chromatograph was online‐coupled to a maXis plus UHR‐QTOF System (Bruker Daltonics) via a CaptiveSpray nanoflow electrospray source. Acquisition of MS/MS spectra after CID fragmentation was performed in data‐dependent mode at a resolution of 60000. The precursor scan rate was 2 Hz processing a mass range between *m*/*z* 175 and 2000. A dynamic method with a fixed cycle time of 3 s was applied via the Compass 1.7 acquisition and processing software (Bruker Daltonics). Prior to database searching with Protein Scape 3.1.3 (Bruker Daltonics) connected to Mascot 2.5.1 (Matrix Science), raw data were processed in Data Analysis 4.2 (Bruker Daltonics). A customized database comprising the sequences of the RGGGPS, RGGGPS_CKr__K144C^−Am^, RGGGPS_OC__W139Y and RGGGPS_OC__W139ONBY^−hv^ proteins as well as common contaminants was used for a database search with the following parameters: enzyme specificity trypsin with two missed cleavages allowed, precursor tolerance of 10 ppm, and MS/MS tolerance of 0.04 Da. As general variable modifications were included: deamidation of asparagine and glutamine, oxidation of methionine, and carbamidomethylation or propionamide modification of cysteine. BrEtAm induced modification of cysteine resulting in the lysine mimetic Lys‐γ‐S, was detected as a customized variable modification of lysine. ONBY was detected as 2‐nitrobenzyl modification, reduced ONBY as 2‐aminobenzyl modification of tyrosine. MS/MS spectra of the specific modifications were inspected manually.

## Conflict of interest

The authors declare no conflict of interest.

## Supporting information

As a service to our authors and readers, this journal provides supporting information supplied by the authors. Such materials are peer reviewed and may be re‐organized for online delivery, but are not copy‐edited or typeset. Technical support issues arising from supporting information (other than missing files) should be addressed to the authors.

Supporting Information
