# Supplementary material for: Controlling Enzymatic Activity by Modulating the Oligomerization State via Chemical Rescue and Optical Control
Source: Chembiochem. 2021 Oct 22;23(5):e202100490. doi: 10.1002/cbic.202100490 (PMC9298306; doi:10.1002/cbic.202100490)
Supplement: Supplementary file 1 — Supporting Information [file CBIC-23-0-s001.pdf]

ChemBioChem

Supporting Information

Controlling Enzymatic Activity by Modulating the Oligomerization State via Chemical Rescue and Optical Control

Cosimo Kropp, Astrid Bruckmann, and Patrick Babinger*

Table of Contents

	page
Table S1. Oligomerization states and molecular weights of GGGPS complexes.	S2
Table S2. Primer sequences for production of the substitutions by site directed mutagenesis.	S3
Figure S1. Analytical SEC of N4 and N12.	S4
Figure S2. Steady-state kinetic measurement of AncGGGPS_N12.	S5
Figure S3. Activity test of GGGPS variants N4 and RGGGPSOC_W139ONBY^{-hv}.	S5
Figure S4. Chemical structures of modified amino acids used in this study.	S6
Figure S5. LC-MS/MS analysis of GGGPS variants.	S7
Figure S6. Reactivation of RGGGPS$_{CKr}$_K144C.	S8
Figure S7. Steady-state kinetic measurements…	S9
Figure S8. Structural integrity of GGGPS variants characterized in this study.	S10
Figure S9. Analysis of substrate binding to GGGPS variants.	S11
Figure S10. Thermal stability of GGGPS variants followed by nanoDSF.	S12
References	S13

Table S1. Oligomerization states and molecular weights of GGGPS complexes.[a]

protein [peak][b]	V_E (mL)	MW^{calc} (kDa)	SLS MW^{app} (kDa)	oligo. state
mtGGGPS	12.9	159.15	151.5	hexamer
$mtGGGPS_{C194A}$	12.9	158.96	162.2	hexamer
$mtGGGPS_{C194A}$_K146C^{-Am}	15.1	52.94	55.0	dimer
$mtGGGPS_{C194A}$_K146C^{+Am}	13.0	160.0	178.3	hexamer
N4	15.1	53.55	57.1	dimer
N12	13.0	160.38	157.6	hexamer
N4_IF_n12 (=RGGGPS)	13.0	161.48	158.6	hexamer
$RGGGPS_{CKr}$_K144C^{-Am}	15.2	53.64	54.8	dimer
$RGGGPS_{CKr}$_K144C^{+Am} [1]	13.1	162.15	167.0	hexamer
$RGGGPS_{CKr}$_K144C^{+Am} [2]	15.2	53.64	54.8	dimer
$RGGGPS_{CKr}$_K144C^{+OH}	15.1	53.64	56.5	dimer
$RGGGPS_{OC}$_W139Y [1]	13.0	160.94	152.5	hexamer
$RGGGPS_{OC}$_W193Y [2]	15.1	53.65	48.9	dimer
$RGGGPS_{OC}$_W139ONBY^{-hv}	15.1	53.95	50.3	dimer
$RGGGPS_{OC}$_W139ONBY^{+hv} [1]	13.0	160.94	149.7	hexamer
$RGGGPS_{OC}$_W139ONBY^{+hv} [2]	15.1	53.95	50.3	dimer

[a] The elution profiles are shown in Figure 3 and Figure S1. V_E, elution volume obtained in analytical gel filtration chromatography; MW^{calc}, MW calculated from the amino acid sequence for dimer or hexamer; SLS MW^{app}, apparent MW as deduced from SLS data; oligo.state, deduced oligomerization state. Characterization of mtGGGPS, N4, N12, and N4_IF_n12 has been performed previously,[1] but has been repeated for this publication.

[b] When two peaks eluted, peaks are labelled as [1] and [2].

Table S2. Oligonucleotide sequences for production of the substitutions by site-directed mutagenesis.

name	forward primer	reverse primer
mtGGGPS_C194A	CCACAGATCAGATACTCATAGTTGGCG	CTCGCTTAACAAGGGCTATCATCTC
mtGGGPS_K146C	CCGGTCCCCAGGAACAAACC	GCAGGTGTCACCGACCCATCC
RGGGPS_K144C	CCTATTCCGCGTCATAAACCG	GCATGCATCACCAACCCAACC
RGGGPS_W139Y	GTTGGTGATGCAAAACCTATTCCG	ATAACCAACGGTTTCACCCGGTTC
RGGGPS_W139amber	GTTGGTGATGCAAAACCTATTCCGC	CTAACCAACGGTTTCACCCGGTTC

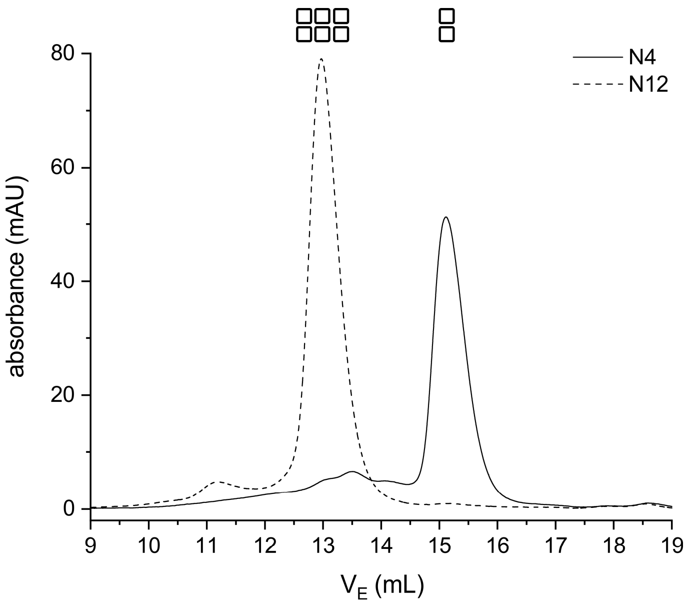

Figure S1. Analytical SEC of N4 and N12. 40 µM of protein (subunit concentration) was applied to a S200 10/300 GL analytical column, which has been equilibrated with 50 mM potassium phosphate, pH 7.5, 300 mM KCl. Elution was performed at a flow rate of 0.4 ml/min, followed by measuring the absorbance at 280 nm and plotted against the elution volume. The derived oligomerization state is indicated by symbols. SEC with N4 and N12 have been performed previously,[1] but have been repeated for this publication.

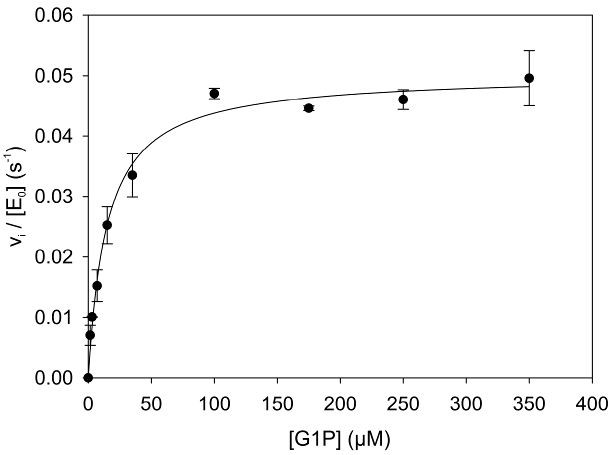

Figure S2. Steady-state kinetic measurement of AncGGGPS_N12. Saturation curves were recorded at 40 °C using a photometric assay, as described in Materials and Methods. The experiment was done in duplicates, the error bars show standard deviations. Kinetic constants were obtained by fitting the Michaelis-Menten equation to the data using SigmaPlot 13.0. The derived parameters are: k_{cat}: 0.05±0.001 s^{-1}, K_M: 14.7±1.6 μM, k_{cat}/K_M: 0.34 x10^4 s^{-1} M^{-1}.

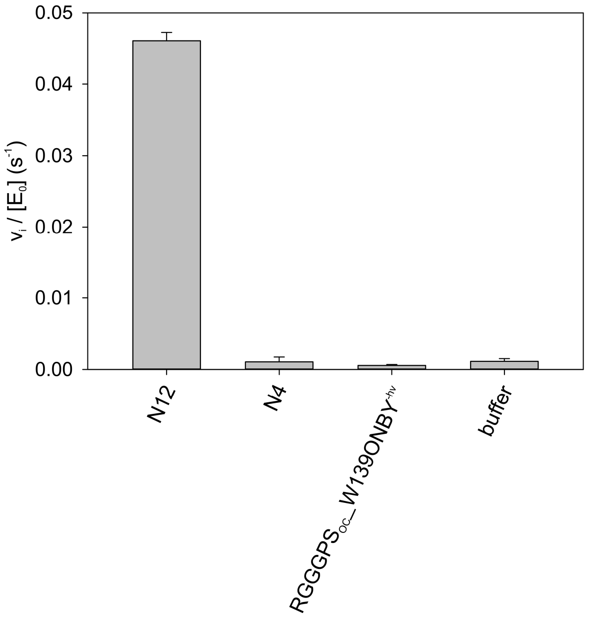

Figure S3. Activity test of GGGPS variants N4 and $RGGGPS_{OC}$_W139ONBY^{-hv}. The variants were assayed at 40 °C at fixed concentrations of 250 μM G1P and 11 μM GGPP, as described for steady-state kinetic measurements. N12 served as reference, buffer was used as control. Note that due to phosphate contaminations in the different components of the assay, there is a detectable background activity in the buffer control that resembles the detected activity in N4 and $RGGGPS_{OC}$_W139ONBY^{-hv}.

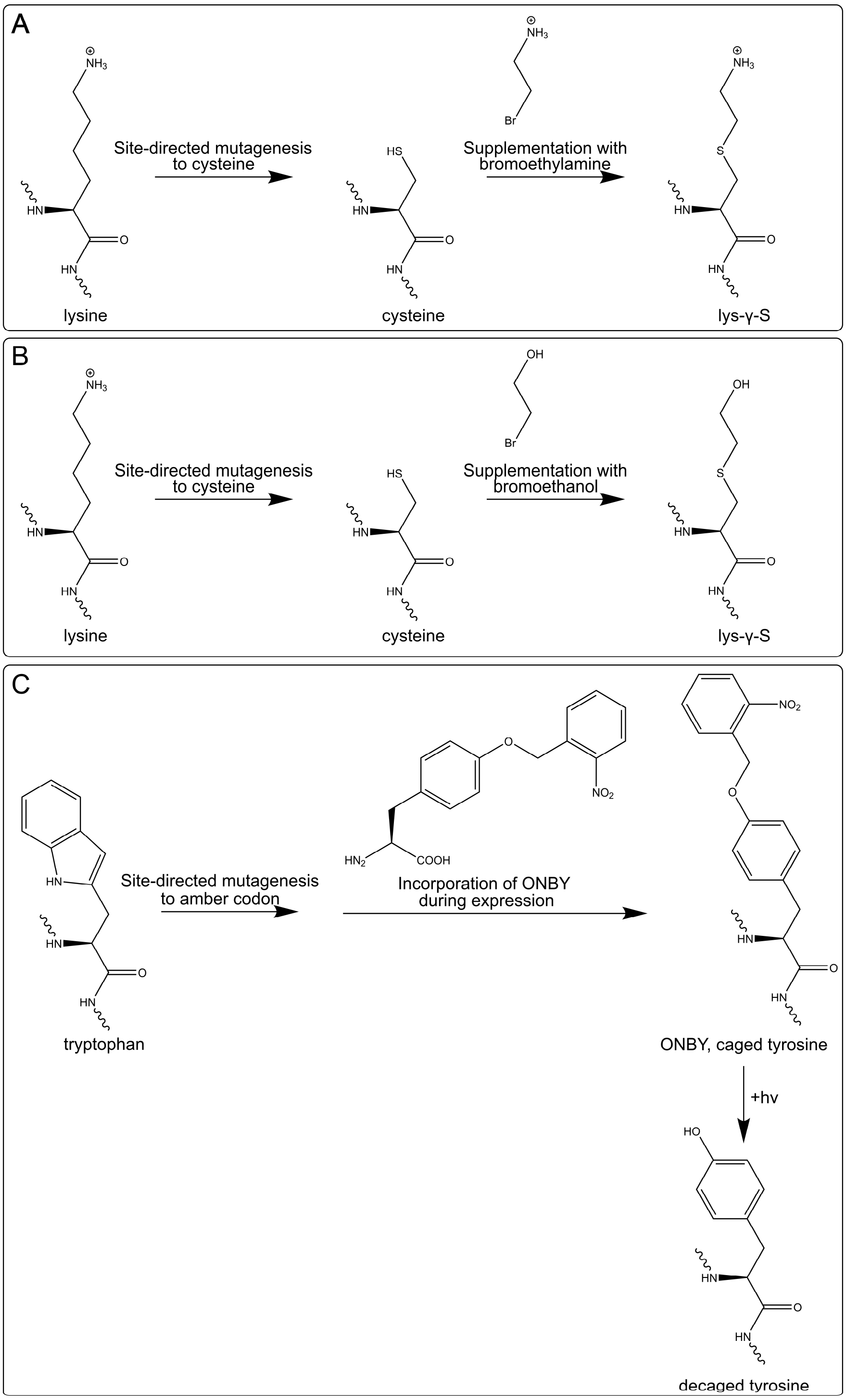

Figure S4. Chemical structures of modified amino acids used in this study.

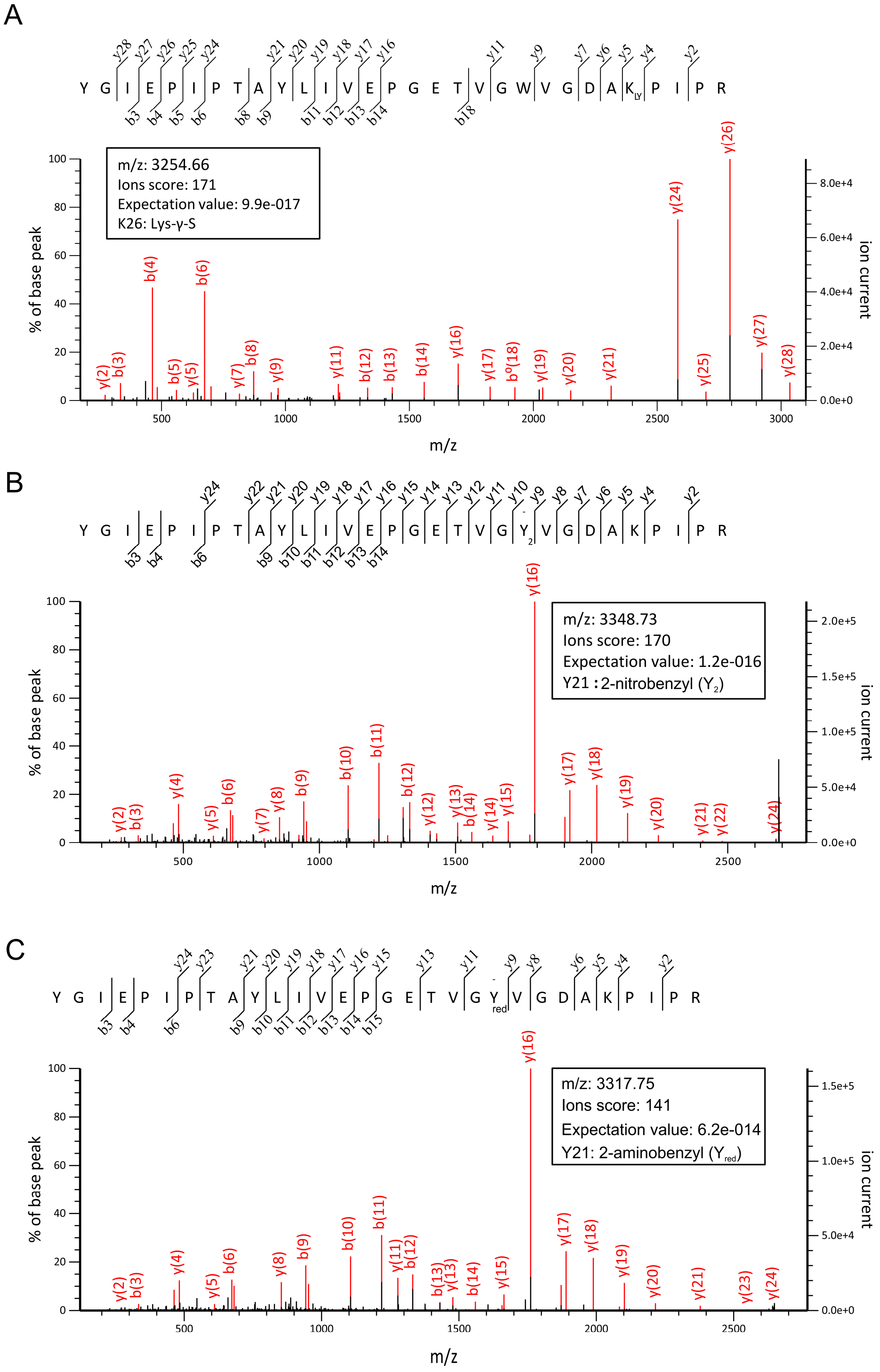

Figure S5. LC-MS/MS analysis of GGGPS variants. MS/MS spectra of tryptic peptides confirming the localization and identity of (A) Lys-γ-S in $RGGGPS_{CKr}$_K144C^{+Am}, as well as (B) ONBY (2-nitrobenzyl-*O*-tyrosine) and (C) reduced ONBY (2-aminobenzyl-*O*-tyrosine) in $RGGGPS_{OC}$_W139ONBY^{-hv}. Fragment ions of the y- and b- series (red peaks) indicate in (A) the presence of Lys-γ-S at position K144 (designated as "K_{LY}"), in (B) the presence of ONBY at position Y139 (designated as "Y_2") and in (C) the presence of reduced ONBY at the same position (designated as "Y_{red}"). Black peaks represent contaminating fragments. The peaks were normalized to the highest signal (100%).

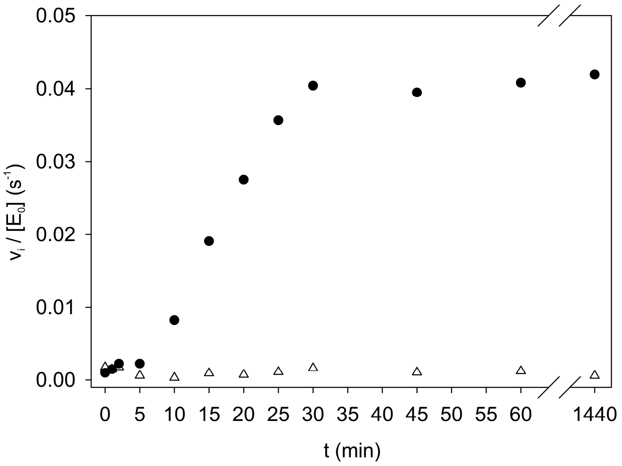

Figure S6. Reactivation of $RGGGPS_{CKr}$_K144C. Enzymatic activity was assayed at 40 °C as described for steady-state kinetic measurements at fixed concentrations of 250 μM G1P and 11 μM GGPP. $RGGGPS_{CKr}$_K144C was incubated without (white triangles) and with 20 mM BrEtAm (black circles) and samples were taken after different times (t = 0, 1, 2, 5, 10, 15, 20, 25, 30, 45, 60, and 1440 min).

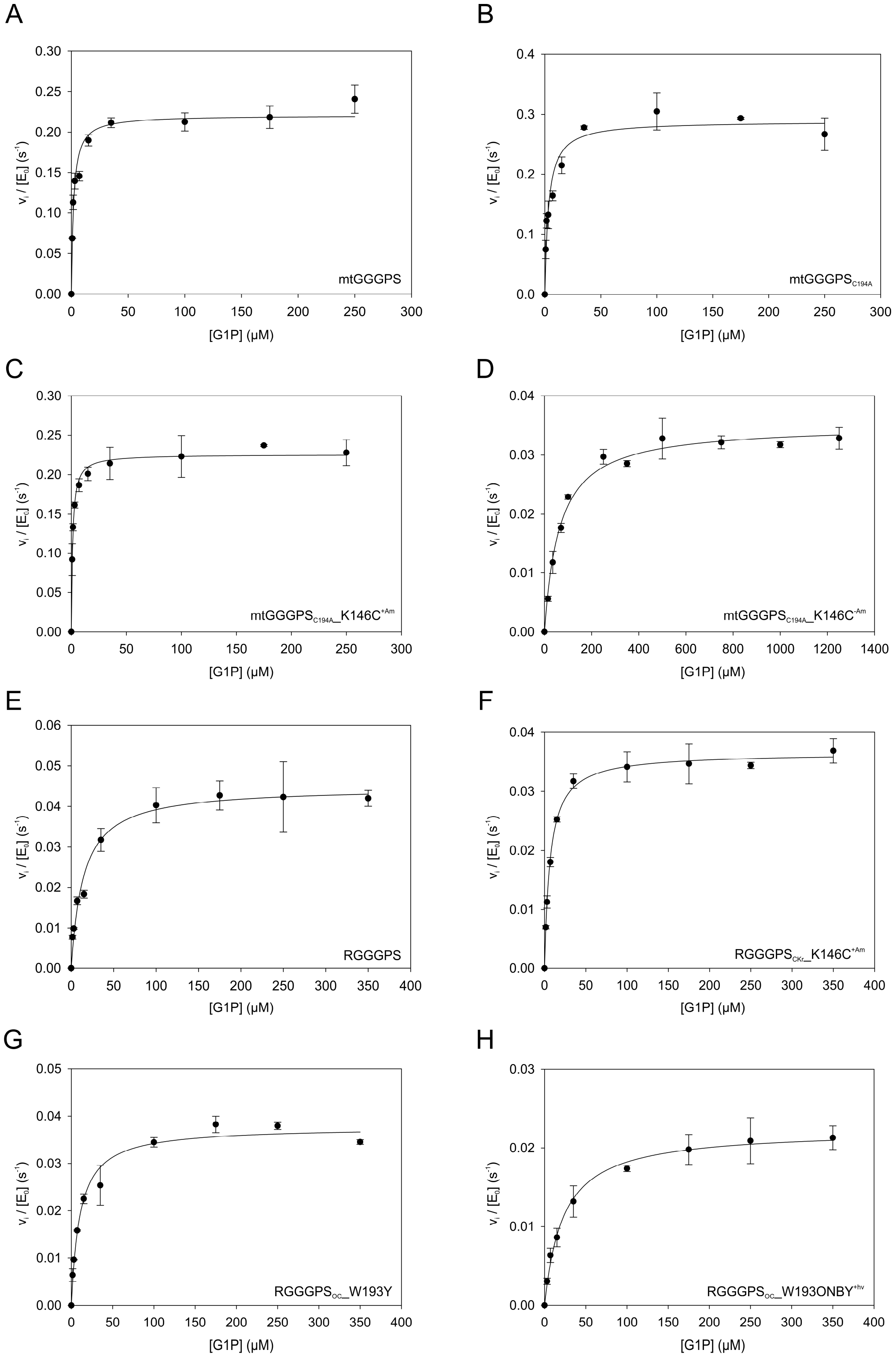

Figure S7. Steady-state kinetic measurements of (A) mtGGGPS wild-type, (B) mtGGGPS_C194A, (C) mtGGGPS$_{C194A}$_K146C^{+Am}, (D) mtGGGPS$_{C194A}$_K146C^{-Am}, (E) RGGGPS (= N4_IF_N12), (F) RGGGPS$_{CKr}$_K144C^{+Am}, (G) RGGGPS$_{OC}$_W139Y and (H) RGGGPS$_{OC}$_W139ONBY^{+hv}. Saturation curves were recorded at 40 °C using a photometric assay, as described in Materials and Methods. The experiment was done in duplicates, the error bars show standard deviations. Kinetic constants were obtained by fitting the Michaelis-Menten equation to the data using SigmaPlot 13.0. The derived parameters k_{cat}, K_M, and k_{cat}/K_M are listed in Table 1.

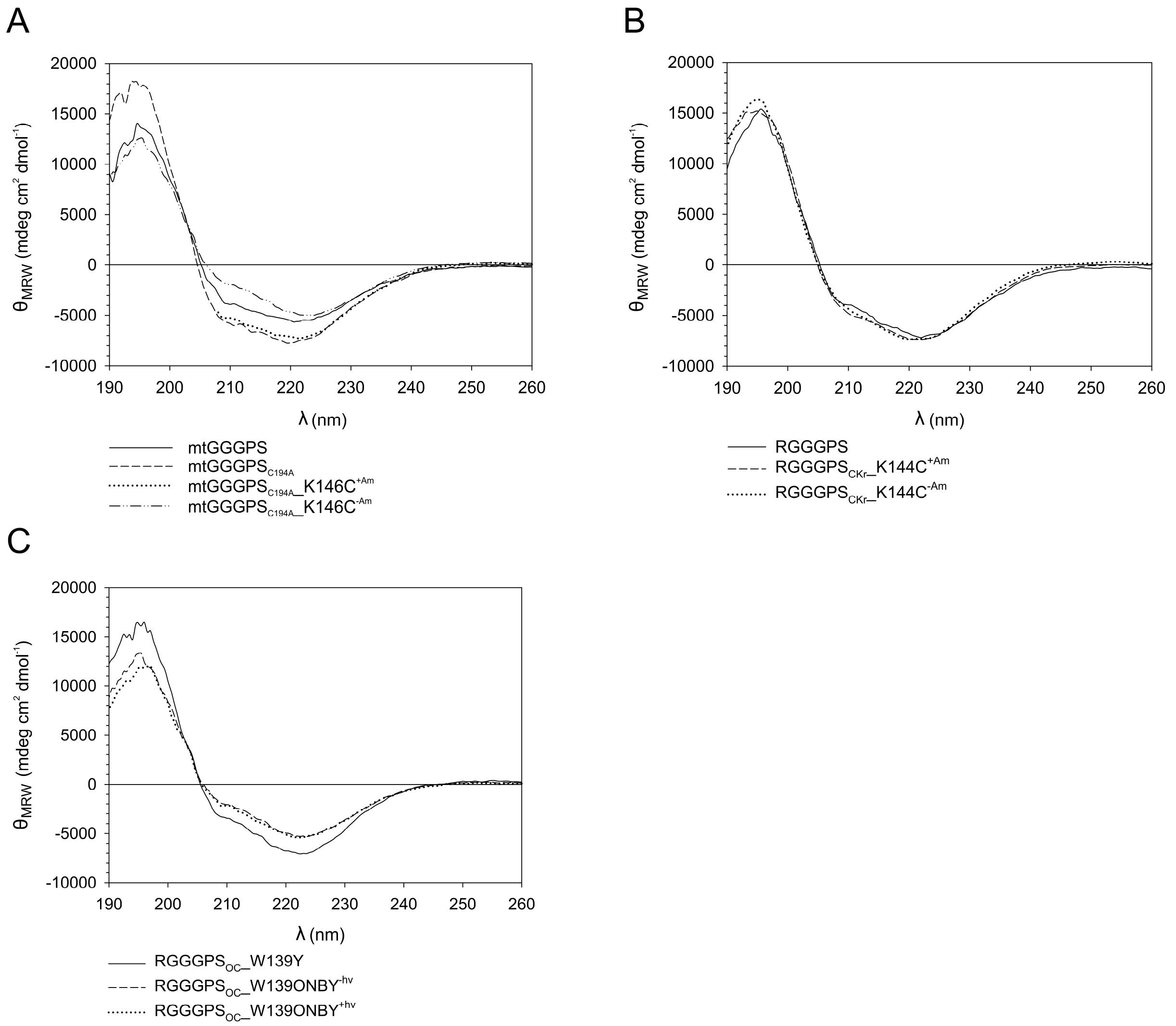

Figure S8. Structural integrity of GGGPS variants characterized in this study. Far-UV CD spectra of all variants (6 µM, subunit concentration) were recorded in 10 mM potassium phosphate, pH 7.5 from 190 nm to 260 nm (d = 1 mm) at 25 °C.

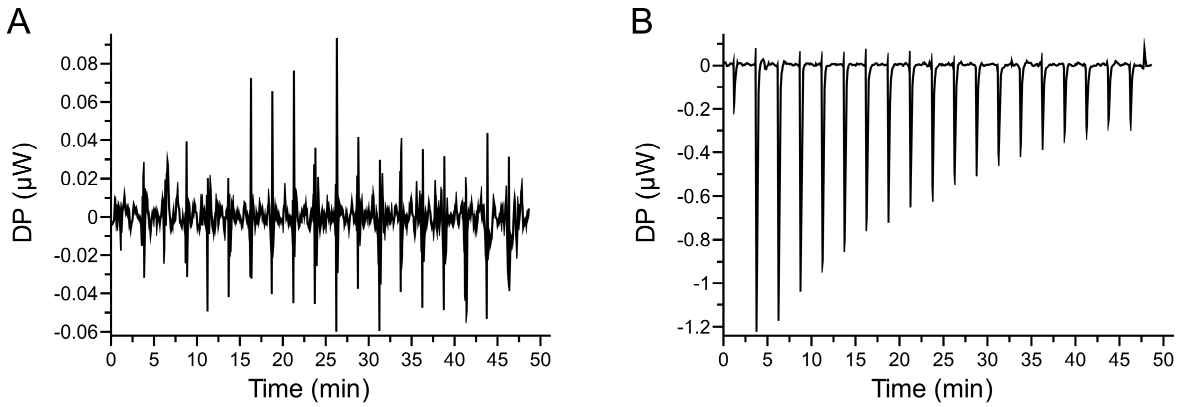

Figure S9. Analysis of substrate binding to GGGPS variants. The binding of the substrate G1P to (A) $RGGGPS_{CKr}$_K144C^{-Am} and (B) $RGGGPS_{CKr}$_K144C^{+Am} was monitored by isothermal titration calorimetry (ITC). G1P was stepwise titrated in 2.5 min intervals to the proteins. The differential power between reference and sample cell (DP) is plotted against the time.

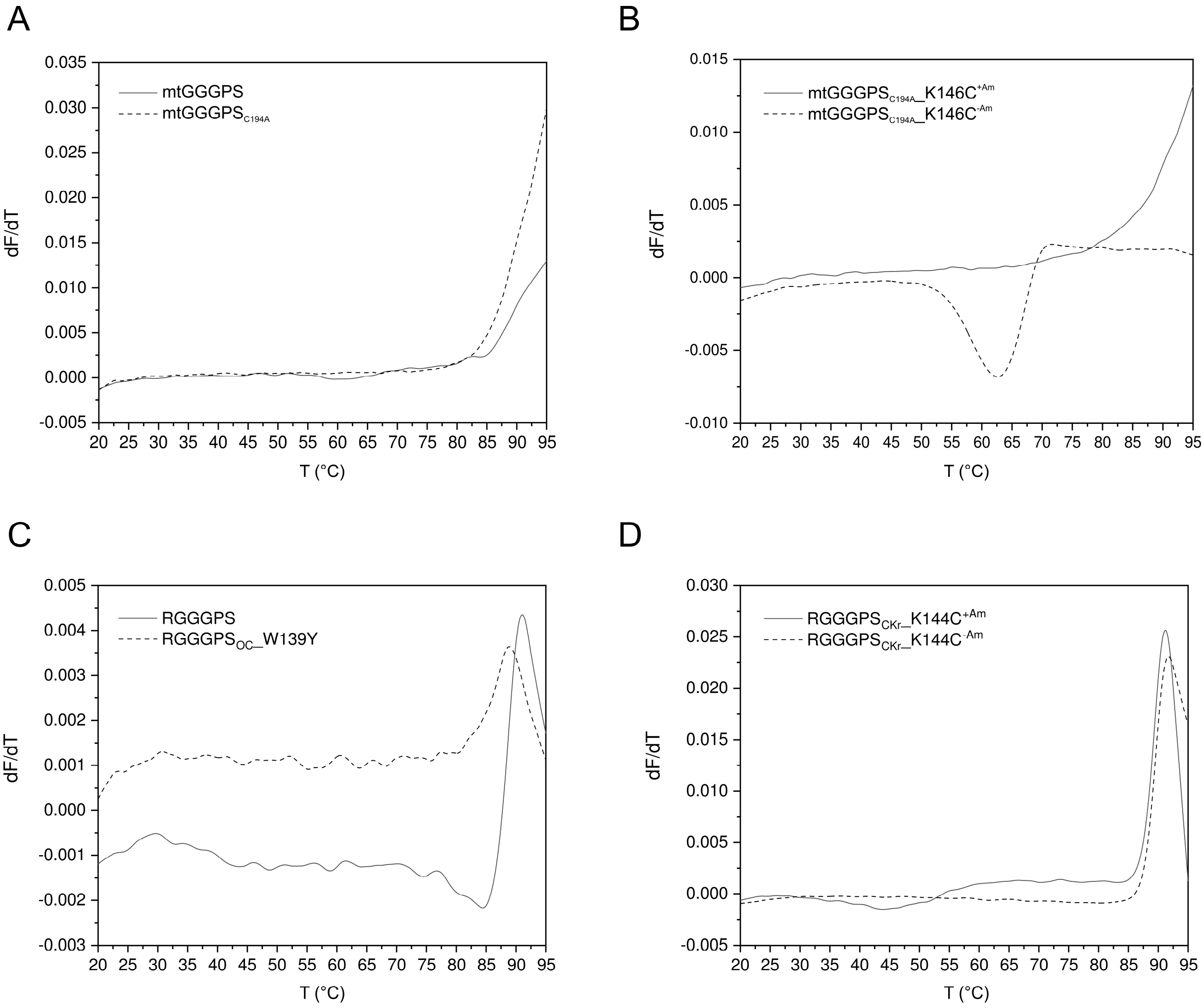

Figure S10. Thermal stability of GGGPS variants followed by nanoDSF. The change in the ratio of the fluorescence emission at 350 and 330 nm of (A and B) 20 µM or (C and D) 40 µM protein (subunit concentration) in 50 mM potassium phosphate, pH 7.5 was monitored from 20 to 95 °C at a scan rate of 1 K/min, and the first derivative of the fluorescence ratio with respect to temperature was plotted against temperature. Extrema in the curve point to thermal transitions. Note that there is a minimum in the mtGGGPS variant (resulting from a decrease in the F_{350}/F_{330} ratio), but maxima in the RGGGPS variants (resulting from an increase in the F_{350}/F_{330} ratio), because the change in exposure to water upon denaturation is different for those variants.[2]

References

[1] C. Kropp, K. Straub, M. Linde, P. Babinger, *Protein Sci.* **2020**, *30*, 583-596.

[2] M. Linde, K. Heyn, R. Merkl, R. Sterner, P. Babinger, *Biochemistry* **2018**, *57*, 2335-2348.
